# Characteristics of behavioural addiction in Parkinson’s disease patients with self-reported impulse control disorder and controls matched for levodopa equivalent dose: a matched case–control study

**DOI:** 10.1007/s00702-023-02588-8

**Published:** 2023-01-20

**Authors:** Bernd Leplow, Daniela Renftle, Mareike Thomas, Katja Michaelis, Susanne Solbrig, Walter Maetzler, Daniela Berg, Inga Liepelt-Scarfone

**Affiliations:** 1 Department of Psychology, Martin-Luther-University Halle-Wittenberg, Emil-Abderhalden-Str. 26-27, Halle, 06108 Germany; 2 Department of Neurodegenerative Diseases, Hertie Institute for Clinical Brain Research, University of Tübingen, Tübingen, Germany; 3 German Center for Neurodegenerative Diseases (DZNE), University of Tübingen, Tübingen, Germany; 4 Department of Neurology, Christian-Albrechts-University, Kiel, Germany; 5 IB Hochschule für Gesundheit und Soziales, Stuttgart, Germany

**Keywords:** Parkinson’s disease, Impulse control disorder, Personality traits, Premorbid personality traits, Substance use disorder, Behavioural addiction

## Abstract

Impulse control disorders (ICD) in Parkinson’s disease (PD) frequently occur, not always as a direct consequence of dopaminergic medication. This study investigated premorbid personality traits and behavioural characteristics in non-demented PD patients with self-reported symptoms of ICD (PD-srICD). From a total of 200 non-demented PD patients who filled out questionnaires assessing symptoms and severity of ICD, those were classified as PD-srICD (*n* = 32) who reported current occurrence of at least one compulsive behaviour (gambling, sexual behaviour, buying behaviour, or eating). As a control group, 32 patients with no self-reported ICD symptoms were matched for levodopa equivalent daily dose. The demographic, clinical, and premorbid personality profiles were compared between both groups. Frequency of psychological characteristics indicating substance use disorder was evaluated in patients with PD-srICD. Patients with PD-srICD were more frequently male, younger at examination, had earlier PD onset, more depression, higher non-motor burden, less quality of life (*p* < 0.05, respectively), and more frequently reported premorbid sensation seeking/novelty orientation (*p* = 0.03) and joyful experience of stress (*p* = 0.04) than patients in the control group. Of patients with PD-srICD, 90.6% reported at least one behavioural characteristic of substance use disorder, most frequently positive expectations following ICD behaviour and illusional beliefs about its behavioural control. Signs of addiction were common among patients with PD-srICD. Therefore, the profile of psychological characteristics in patients with PD-srICD resembled that of patients with substance use disorder. It can be concluded that dopamine replacement therapy (DRT) alone does not account for PD-srICD and that thorough psychological diagnostics are recommended.

## Introduction

Impulse control disorder (ICD) is defined as one or more behaviours that are performed repetitively, excessively, compulsively, and to an extent affecting the diseased person’s life (Weintraub and Nirenberg 2013). Occurrence and severity of ICD behaviours can range from subclinical level to a stage allowing clinical diagnosis (Baig et al. 2019). Presence and severity of ICD behaviours lead to negative consequences such as disastrous financial problems, loss of employment, severe health problems, decrease in quality of life, and interpersonal and marital conflicts (Ferrara and Stacy 2008; Weintraub and Claassen 2017; Phu et al. 2014). Therefore, the identification of people at high risk for the development of ICD and an early diagnosis are of utmost importance to maintain patients’ quality of life.

ICD in Parkinson’s disease (PD) is a well-known side effect of dopamine replacement therapy (DRT), being most closely related to the use of dopamine agonists (Voon and Fox 2007; De Wit et al. 2022).

Prevalence of ICD behaviours in patients with DRT is around a minimum of 17% (Weintraub et al. 2010) with increase in prevalence during the progression of the disease (Corvol et al. 2018; Marín‐Lahoz et al. 2022). However, occurrence of ICD behaviours is also reported in 3% of patients without DRT (Weintraub et al. 2010), and approximately 20% of newly diagnosed and untreated PD patients report some ICD or related behaviour (Weintraub et al. 2013). Therefore, factors other than DRT might influence vulnerability regarding the development of ICD in PD, arguing for the need of additional risk factors with high predictive value for ICD within the disease course.

Known risk factors for the emergence of ICD behaviours in PD are male sex, younger age, early onset of disease, longer duration of disease and more non-motor symptoms (Weintraub et al. 2013; Cao et al. 2022). Furthermore, pre-PD history of anxiety, depression, substance use disorder, bipolar disorder, pathological gambling, and impulsivity are known to increase future ICD risk (Weintraub and Claassen 2017; Grall-Bronnec et al. 2018). PD patients with first-degree relatives showing these respective disorders are also more prone to develop ICD behaviours following DRT (Weintraub et al. 2006, 2015; Ambermoon et al. 2011), arguing for the contribution of genetic factors on the emergence of ICD in PD.

Regarding life style factors tobacco smoking and alcohol consumption may enhance the risk for ICD development in PD (Marin-Lahoz et al. 2018). Recently, a combination of clinical and genetic markers was able to support clinical diagnosis of ICD behaviours in patients with PD (Weintraub et al. 2022), also supporting the assumption that other factors than DRT might modulate onset and severity of ICD in PD.

ICD behaviours in PD patients often resemble addiction (Probst and van Eimeren 2013; Limotai et al. 2012; e.g., Dagher and Robbins 2009; Voon et al. 2010b, Ramdave et al. 2020). While PD patients without ICD are known to show less sensation seeking/novelty orientation (Djamshidian et al. 2011b) and more harm avoidance (Tomer and Aharon-Peretz 2004), PD patients with ICD behaviours are more prone to risk-taking behaviour and show a preference for uncertainty, more perseverative responses, and a heightened drive to engage in gambling and gambling-related behaviours (Marques et al. 2018). Altered reward learning (Djamshidian et al. 2011a), enhanced risk learning (Djamshidian et al. 2010), and reduced learning of punishments (Leplow et al. 2017) was also described in PD patients with ICD. Moreover, maintaining problem behaviour despite negative consequences, focussing the attention towards addictive stimuli and loss of behavioural control typical for substance use disorder patients are occasionally reported for PD patients with ICD.

To date, it is not known to what extent the profile of ICD in PD resembles that seen in substance use disorder. Thus, the aim of this study was twofold: first, we investigated whether the premorbid personality factors typical for people who are at risk for substance use disorder also characterize those PD patients with self-reported ICD symptoms (PD-srICD). Second, we wanted to know to which extent the behavioural characteristics of patients with substance use disorder can also be found in PD-srICD patients.

## Methods

### Patients

Data were collected within the frame of the “Amyloid-Beta in cerebrospinal fluid as a risk factor for cognitive dysfunction in Parkinson’s disease (ABC-PD)” study. Patients were recruited between March 2014 and December 2017 from the ward and outpatient clinic of the Neurology Department of the University Hospital in Tübingen as well as through contacts with neurologists practicing in the near region. Details regarding patient selection, inclusion and exclusion criteria have been described previously (Becker et al. 2020). In brief, 200 patients with PD diagnosis according to the UK Brain Bank criteria (Hughes et al. 1992) who agreed to a lumbar puncture, were aged between 50 and 85 years, able to give written informed consent, and had no concomitant diseases interfering with cognition were included. No patient had diagnosis of PD dementia (Emre et al. 2007), deep brain stimulation, or previous alcohol or drug abuse. For the presented analysis, inclusion criteria were treatment with DRT as well as consent to filling out the additional questionnaire about history of ICD and premorbid personality traits. The study was approved by the Ethics Committee of the Medical Faculty (protocol nr. 686/2013BO1).

Out of 200 patients eligible for data analysis, 32 (16%) PD-srICD patients were identified. Patients met criteria for PD-srICD if they reported occurrence of at least one out of the compulsive behaviours gambling, sexual behaviour, buying behaviour or eating within the last four weeks (assessed by the Questionnaire for Impulsive-Compulsive Disorders in Parkinson’s Disease Short version; QUIP-S; part score ≥ 1) (Weintraub et al. 2009) and judged the urge to perform these behaviours as increased after PD diagnosis (assessed by a second independent questionnaire). To compare the clinical profile and premorbid personality traits, control patients (PD-CO) who reported no compulsive gambling, sexual, buying or eating behaviours were matched by their levodopa equivalent daily dose (LEDD). Mean difference in LEDD between corresponding pairs was 39.1 mg (*SD* = 50.1), maximum difference was 116 mg and minimum difference 0 mg.

### Assessments

Demographic data like gender, age at PD onset, and disease duration were assessed. LEDD was calculated according to Tomlinson et al. (2010) with additional consideration of opicapone (levodopa dose *0.5) and safinamide (LEDD = 100 mg) intake (Schade et al. 2020).

Severity of motor impairment was assessed by the Movement Disorder Society Unified Parkinson’s Disease Rating Scale Part III (MDS-UPDRS-III) (Goetz et al. 2008) including the Hoehn and Yahr score (Goetz et al. 2008). The Montreal Cognitive Assessment (MoCA) was used to screen for global cognitive status (Nasreddine et al. 2005). Nonmotor burden was assessed with the German versions of the Non-Motor Symptoms Questionnaire (PD-NMS-Q) (Storch et al. 2010) and the Beck Depression Inventory-II (BDI-II) (Hautzinger et al. 2009).

Health-related quality of life (HRQoL) was investigated using the Parkinson’s Disease Questionnaire (PDQ-39) (Peto et al. 1998). The 39 items (rating: 0 = never to 4 = always) of the PDQ-39 are divided into 8 domains: mobility (10 items), activities of daily living (6 items), emotional well-being (6 items), stigma (4 items), social support (3 items), cognition (4 items), communication (3 items), and bodily discomfort (3 items). A summary index and the subscores of each domain were calculated according to the official scoring algorithm. Higher scores indicate lower HRQoL.

By means of a self-developed questionnaire, participants were assessed with respect to their ICD history and premorbid personality factors associated with a risk to develop substance use disorder later in life. Patients were asked whether specific ICD-related behaviours had increased following the administration of DRT and whether the occurrence of such behaviours had an impact on their daily lives and that of their caregivers. Further items were related to onset and course over time of the ICD-related behaviours, e.g., “When did the impulsive behaviour occur first?” and “Do you feel pleasure when you give in to the urge?”. The premorbid personality characteristics sensation seeking/novelty orientation (“Did you like to participate in new activities”?) and pleasurable stress experience (“Did you usually experience pleasure in high-stress situations?”) as well as premorbid psychological burden severe enough to seek for medical or psychological treatment were inquired. Premorbid personality traits were explicitly related to the time prior to PD diagnosis.

For the assessment of behavioural ICD characteristics we adapted an 11 item questionnaire for the diagnosis of addictive behaviour (Grüsser and Thalemann 2006). For instance, it was asked for positive expectations following ICD behaviour, illusional beliefs about its behavioural control, and maintenance of ICD behaviour despite negative consequences. The total number of signs indicative of substance use disorder was calculated for each PD-srICD patient.

To further characterize ICD, the number of reported ICD symptoms (part score ≥ 1) in QUIP-S part A to D was calculated for each patient. The QUIP-S subscore for part A to D and the QUIP-S total score were also evaluated (Weintraub et al. 2009).

### Statistical analysis

Clinical data were collected and managed using REDCap electronic data capture tools hosted at the Hertie Institute for Clinical Brain Research (Harris et al. 2009). Data were analysed using the statistical computing software R (Version 4.1.2) (R Core Team 2021) with all alpha levels set to 0.05, two-tailed. Non-categorical variables were tested for normal distribution with Shapiro–Wilk tests. Parametric and non-parametric statistics are reported as appropriate. Between-group differences in demographics and QUIP-S data were analysed using Chi-square test (categorical variables), Mann–Whitney *U* test (non-parametric variables) or two-sample *t* test for unpaired samples (parametric variables).

Further comparisons between study groups were corrected for identified demographic confounders: logistic regression analyses were performed with group status as dependent variable, the variable of interest as independent variable, and gender, age at examination, and age at PD onset as confounders. Non-parametric variables were transformed to meet model requirements for logistic regression analysis. As measure of model fit, *pseudo-R*^*2*^ was calculated according to Nagelkerke (1991). Correlation coefficients between the total number of signs indicative of substance use disorder and QUIP-S data were calculated based on Spearman rank correlation analysis.

## Results

Out of the total sample of 200 patients who completed the questionnaire, 32 (16%) meet criteria for classification as PD-srICD. Regarding QUIP-S part A to D, abnormal sexual behaviour (*n* = 23, 71.9%) was the most frequently reported ICD symptom in patients with PD-srICD, followed by compulsive eating (*n* = 17, 53.1%), buying (*n* = 5, 15.6%) and gambling (*n* = 2, 6.2%). The median number of compulsive symptoms reported by PD-srICD patients in the QUIP-S part A to D was 1 (*range*: 1 to 3).

In general, patients classified as PD-srICD were more frequently male (*p* < 0.01), younger at examination (*p* < 0.01) as well as younger at time of PD onset (*p* < 0.01) compared to the control group matched according to the total LEDD (see Table 1 for details). Dosage of dopaminergic medication types as well as severity of PD symptoms assessed by the UPDRS-III and Hoehn and Yahr score did not differ between both groups (*p* > 0.05, respectively). None of the patients in both groups had intake of anticholinergic drugs.

**Table 1:** Characteristics of the total Parkinson’s disease (PD) sample, the control group (PD-CO) and the group with self-reported symptoms of impulse control disorder (PD-srICD)

	Total	PD-CO	PD-srICD	*p* value
*N*	64	32	32	
Male gender^a^	44 (68.8)	16 (50.0)	28 (87.5)	** < 0.01**
Age at examination^b^, years	64.9 (7.9)	67.7 (8.3)	62.1 (6.5)	** < 0.01**
Education, years	13.0 (8.0–21.0)	13.0 (8.0–19.0)	13.0 (11.0–21.0)	0.39
Disease duration, years	5.5 (0.7–20.6)	5.0 (0.9–13.3)	6.1 (0.7–20.6)	0.48
Age at disease onset^b^, years	58.8 (9.4)	62.0 (9.9)	55.5 (7.8)	** < 0.01**
Hoehn and Yahr^a^				0.80
1	7 (10.9)	3 (9.4)	4 (12.5)	
2	43 (67.2)	21 (65.6)	22 (68.8)	
> 2	14 (21.9)	8 (25.0)	6 (18.7)	
MDS-UPDRS-III	26.0 (3.0–66.0)	24.5 (8.0–64.0)	28.5 (3.0–66.0)	0.36
LEDD	657.5 (210.0–1744.8)	657.5 (240.0–1668.0)	644.5 (210.0–1744.8)	0.99
Levodopa use^a^	44 (68.8)	24 (75.0)	20 (62.5)	0.28
LED levodopa	200.0 (0.0–1100.0)	212.5 (0.0–800.0)	150.0 (0.0–1100.0)	0.39
DA use^a^	52 (81.3)	25 (78.1)	27 (84.4)	0.52
LED DA	200.0 (0.0–600.0)	180.0 (0.0–480.0)	209.5 (0.0–600.0)	0.42
COMT inhibitors use^a^	21 (32.8)	11 (34.4)	10 (31.3)	0.79
MOA-B use^a^	35 (54.7)	16 (50.0)	19 (59.4)	0.45
NMDA use^a^	15 (23.4)	7 (21.9)	8 (25.0)	0.77
BDI-II	9.5 (1.0–27.0)	8.0 (1.0–27.0)	11.5 (1.0–24.0)	** 0.04**^cd^
PD-NMS-Q	8.5 (0.0–23.0)	7.5 (0.0–18.0)	10.0 (0.0–23.0)	** < 0.01**^c^
FAQ	1.0 (0.0–17.0)	1.0 (0.0–8.0)	2.0 (0.0–17.0)	0.16^cd^
MoCA	27.0 (16.0–30.0)	27.0 (16.0–30.0)	26.5 (16.0–30.0)	0.80^ce^
PDQ-39 summary index	19.0 (1.0–75.0)	16.5 (1.0–42.0)	21.0 (6.0–75.0)	0.052^cd^
- Mobility	13.8 (0.0–80.0)	12.5 (0.0–77.5)	17.5 (0.0–80.0)	0.29^cd^
- Activities of daily living	20.8 (0.0–79.2)	20.8 (0.0–62.5)	27.1 (4.2–79.2)	0.32^cd^
- Emotional wellbeing	16.7 (0.0–75.0)	8.3 (0.0–50.0)	25.0 (0.0–75.0)	0.07^cd^
- Stigma	9.4 (0.0–75.0)	6.3 (0.0–75.0)	12.5 (0.0–75.0)	0.17^cd^
- Social support	8.3 (0.0–100.0)	0.0 (0.0–66.7)	8.33 (0.0–100.0)	0.56^cd^
- Cognition	18.8 (0.0–75.0)	18.8 (0.0–62.5)	25.0 (0.0–75.0)	** 0.047**^cd^
- Communication	25.0 (0.0–75.0)	8.3 (0.0–75.0)	33.3 (0.0–75.0)	** 0.045**^c^
- Bodily discomfort	16.7 (0.0–75.0)	16.7 (0.0–75.0)	16.7 (0.0–75.0)	0.19^cd^

If not otherwise indicated, values are given as *Mdn* (*range*); significant p-values are highlighted in bold

^a^ = values given as *n* (*percentage*)

^b^ = values given as *M* (*SD*)

^c^ = Binary logistic regression analysis with group as dependent variable, (transformed) variable of interest as independent variable and gender, age at examination, and age at disease onset as confounders

^d^ = transformation sqrt(x)

^e^ = transformation sqrt(max(x)-x)

*BDI-I *Beck Depression Inventory-II, *COMT* catechol-o-methyltransferase, *DA* dopamine agonist, *FAQ* Functional Activities Questionnaire, *LED* levodopa equivalent dose, *LEDD* levodopa equivalent daily doses, *MDS-UPDRS*-*III* Movement Disorder Society Unified Parkinson’s Disease Rating Scale Part III, *MOA-B* monoamine oxidase-b inhibitors, *MoCA* Montreal Cognitive Assessment, *NMDA* N-methyl-D-aspartate receptor antagonists, *PD-NMS-Q* Parkinson’s Disease Non-Motor Symptom Questionnaire, PDQ-39 Parkinson’s Disease Questionnaire

### Between-group comparison of clinical characteristics and premorbid personality traits

Besides the compulsive behaviours used to define patients group status, excessive hobbyism (QUIP-S part E) was also more frequent in patients with PD-srICD (*n* = 18, 56.2%) compared to PD-CO (*n* = 7, 21.9%, *p* < 0.01). Walking or driving with no intended goal was only reported in patients with PD-srICD (*n* = 4, 12.5%). Most interestingly, frequency of punding (PD-srICD: *n* = 7, 21.9% vs. PD-CO: *n* = 4, 12.5%, *p* = 0.32) as well as the urge to overdose medication intake (QUIP-S part F; PD-srICD: *n* = 3, 9.4% vs. PD-CO: *n* = 5, 15.6%, *p* = 0.45) did not differ between both groups.

Patients with PD-srICD reported a higher number of non-motor symptoms in the PD-NMS-Q (*p* < 0.01) and more severe symptoms of depression in the BDI-II (*p* = 0.04) than the PD-CO group, irrespective of confounding variables. Higher values in the PDQ-39 subscores cognition and communication (*p* < 0.05, respectively) in patients with PD-srICD compared to PD-CO indicated lower HRQoL related to these domains (see Table 1).

**Fig. 1:**
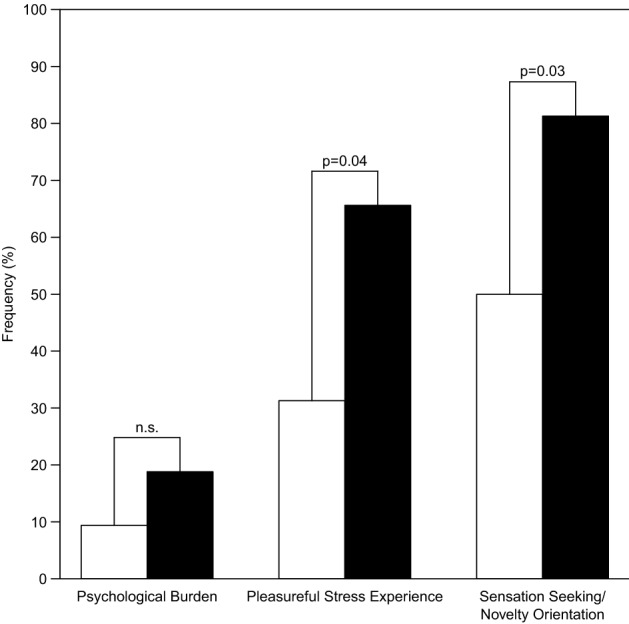
Between-group comparison of Parkinson’s disease patients with (black bars) vs. without (white bars) self-reported symptoms of impulse control disorder regarding premorbid personality traits; *p* values refer to results of logistic regression analyses corrected for the between-group confounders gender, age at examination, and age at disease onset

Premorbid sensation seeking/novelty orientation (*z* = 2.2, *p* = 0.03) was significantly more frequent in patients with PD-srICD compared to the PD-CO group (*pseudo-R*^*2*^ = 0.39; see Fig. 1). Significantly more patients with PD-srICD than PD-CO patients (*z* = 2.0, *p* = 0.04) premorbidly experienced pleasure when being exposed to stressful situations (*pseudo-R*^*2*^ = 0.43; two missing values). The rate of premorbid psychological burden (*pseudo-R*^*2*^ = 0.34; one missing value) and premorbid mental disorders (pseudo-R^2^ = 0.65; 15 missing values) did not differentiate between groups (*p* > 0.05, respectively).

### Characteristics of ICD and substance use disorder in patients with PD-srICD

In total, 90.6% of patients with PD-srICD reported at least one characteristic indicative of substance use disorder. Median number of signs indicative of substance use disorder was 5 (*range*: 1 to 9; three missing values). Especially positive expectations following ICD behaviour, illusional beliefs about its behavioural control, positive emotions following ICD behaviour, and maintenance of ICD behaviour despite negative consequences were frequent in PD-srICD patients (see Table 2 for details).

**Table 2:** Percentage of addictive behaviours in Parkinson’s disease patients with self-reported symptoms of impulse control disorder (srICD, *n* = 32)

Characteristic of substance use disorder	Frequency, %
Positive expectations following srICD behaviour	75.0
Illusional beliefs about behavioural control on srICD excesses [2]	65.6
Positive emotions following srICD behaviour	62.5
Maintenance of srICD even in the case of negative consequences [2]	56.3
Increasing number of stimuli elicit srICD	50.0
Negative feelings if srICD cannot be displayed	40.6
Displaying srICD in the case of negative emotions	40.6
Positive consequences at the beginning turned to negative consequences	31.3
Everyday life is determined by srICD behaviours	25.0
Intensity and frequency of srICD behaviour gradually increase [1]	21.9
Thoughts nearly completely focus on srICD behaviour	21.9

Number of missing values is reported in brackets

Correlation analysis showed that the total number of signs indicative of substance use disorder was significantly correlated with the number of reported ICD symptoms in QUIP-S part A to D (*rho* = 0.64, *p* < 0.001) and the QUIP-S subscore for part A to D (*rho* = 0.47, *p* < 0.01). The QUIP-S total score (*rho* = 0.48, *p* < 0.01) was also significantly correlated with the total number of signs indicative of substance use disorder.

For patients with PD-srICD, first occurrence of compulsive behaviours (gambling, sexual behaviour, buying behaviour or eating) was *Mdn* = 3 years (*range*: 1 to 15) before the study examination date. Seventeen (53.1%) PD-srICD patients had the feeling that their ICD behaviours were related to DRT start. Out of this subgroup, 1 (5.9%) patient reported immediate onset of at least one ICD symptom after DRT start and 6 (35.3%) patients reported ICD symptom onset within four weeks after DRT start.

Twenty-five percent of patients with PD-srICD reported that the extent of their urges became gradually stronger, 12.5% described a rapid symptom increase, 37.5% reported that the urges remained about the same, 9.4% said that the behaviours’ intensities lowered over time, and 15.6% stated that they experienced strong fluctuations of ICD-related behaviours. Half of the PD-srICD patients reported negative consequences for themselves or their caregiver resulting from their urges of compulsive behaviour. In addition, 37.5% of patients with PD-srICD stated that their urges severely interfered with daily activities. The PD-srICD patients described the compulsions as “pleasant” (12.5%), “okay” (28.1%), “irritating” (50.0%) or “not controllable” (9.4%).

## Discussion

Our results show that premorbid risk factors for substance use disorder are more present in PD-srICD compared to PD controls. Especially we found that PD-srICD patients reported higher levels of sensation seeking/novelty orientation prior to PD diagnosis than the control group matched according to total LEDD. Also we found that patients with PD-srICD had experienced premorbid daily hassles as more pleasurable than their LEDD counterparts. Second, we could show that over 90% of PD-srICD patients reported at least one behavioural characteristic indicative of substance use disorder. The number of these signs was correlated to the severity of ICD assessed by the QUIP.

Our results add evidence to the role of specific premorbid personality traits which may increase the risk to develop PD-ICD if the patients have to undergo DRT. Especially the high levels of sensation seeking/novelty orientation seem to be risk factors for the development of ICD-related behaviours. In addition, before onset of Parkinson’s disease PD-srICD patients tended to attribute incentive values to stressful situations. This supports the view that a specific pattern of premorbid personality traits may be responsible for ICD vulnerability following DRT. Thus, our study confirms studies which showed close similarities between substance use disorder and PD-srICD (Probst and van Eimeren 2013; Limotai et al. 2012; e.g., Dagher and Robbins 2009; Voon et al. 2010b). Therefore, our results may reflect the behavioural level of cerebral circuits reward circuits in addiction (Navalpotro-Gomez et al. 2020; Ramdave et al. 2020). This can be explained by role of dopamine transmission in learning.

Thus, phasic dopamine activity is related to learning of rewards, whereas a lack of dopamine activity is associated with avoidance behaviour and learning of punishments (Voon et al. 2010a; Leplow et al. 2017). Moreover, dopamine activity mediates the expectation of positive outcomes following goal-directed behaviour, especially if the outcome probability is about 50%. In these cases, dopamine acts as an enhancer for the incentive value of a discriminative stimulus signalling reward. Phasic dopaminergic responses are also related to positive reward expectations, experience of larger rewards than expected, learning of conditioned stimuli predicting rewards, and active search for these predictive stimuli. These learning behaviours are associated with reduced uncertainty for behavioural changes alongside with sustaining responding to incentive stimuli regardless of their consequences (O’Sullivan et al. 2011). The behaviour of PD-srICD patients was also biased towards reinforcement learning and risk taking behaviour. Taken together, this behavioural profile resembles a behavioural pattern typical for substance use disorder patients.

In this context, it is noteworthy that characteristics of substance use disorder like “maintenance of problematic behaviours despite harmful consequences”, “illusional beliefs about behavioural control”, and “positive expectations following problem behaviours” were seen in the majority of PD-srICD patients. ICD behaviours were conducted even though half of these PD-srICD patients stated to anticipate negative consequences for themselves or their caregiver resulting from their urges. In addition, 37.5% of patients with PD-srICD experienced that their ICD behaviours severely interfered with daily activities. More than half of the PD-srICD patients attributed onset of their ICD behaviours to start of DRT. Thus, our data referring to patient’s retrospective report confirm previous results that dopamine active drugs may enhance the chance of the emergence of ICD-related behaviours in PD patients (Delaney et al. 2012; Voon and Fox 2007; De Wit et al. 2022).

Our study has a number of limitations. Compared to the control group, patients with PD-srICD were more frequently male, younger at the time of examination and displayed a younger age of Parkinson’s disease onset. Moreover, we found a higher number of non-motor symptoms, more severe symptoms of depression, and worse PDQ-39 subscores for cognition and communication. But despite these differences, our basic results are in accordance with the literature (e.g., Weintraub and Claassen 2017; Weintraub et al. 2006, 2010). Moreover, the study sample was carefully selected from a large sample of 200 non-demented patients with confirmed diagnosis of PD according to consensus guidelines. Furthermore, no participant had concomitant diseases affecting cognition. Patients with Deep Brain Stimulation were also excluded as well as those with a history of substance use disorder. Each participant agreed to lumbar puncture and was fully aware of the scope of the study. From this well-defined sample we identified 32 patients showing PD-srICD who could be matched to 32 PD patients without PD-srICD with respect to daily LEDD. Unfortunately, it was not possible to also match with respect to disease duration, but as Table 1 shows, important clinical variables do not differ between groups. Another limitation is related to the retrospective nature of the assessments about the premorbid personality traits, but assessment of PD-srICD was done by careful history taking and a formalized questionnaire.

Formalized PD-srICD assessment was performed using the QUIP, which is a validated questionnaire for the assessment of ICD symptoms in PD so we feel sure that we have obtained valid responses not only with respect to the actual behavioural symptoms during DRT, but also with respect to reports about the patient’s premorbid situation. But further research should add control groups comprised of patients with substance use disorder and verified behavioural addiction such as pathological gambling.

As a consequence of this investigation, it can be concluded that DRT alone does not sufficiently account for the emergence of PD-srICD. Like in substance use disorder, personality traits like sensation seeking and novelty orientation increasing the risk of ICD if DRT have to be administered. Moreover, important similarities between PD-srICD and substance use disorder patients occur. These similarities are related to behavioural characteristics following DRT. This should be considered in diagnostic routine. Furthermore, if DRT-related side effects lead to a behavioural profile which is closely similar to that seen in addiction disorders, psychological treatments should be designed alongside with pharmacological interventions. The psychologic treatment should be designed to target behavioural addictions as has been successfully done by Okai et al. (2013) for PD-ICD patients. This approach included behaviour analysis, motivational analysis, education, treatment of depression, anxiety, dysfunctional stress responses, reorganisation of reinforcement systems, executive functions, and relapse prevention. Overall, psychological attempts in treating PD-ICD resemble those used in the treatment of substance use disorders (see also Jiménez-Murcia et al. 2012). Such a treatment should be addressed to subgroups of patients with uncontrollable dopaminergic side effects which cannot be adequately managed by medical treatment options.

## Data Availability

Data will be available on reasonable request.

## References

[CR1] Ambermoon P, Carter A, Hall WD, Dissanayaka NNW, O'Sullivan JD (2011). Impulse control disorders in patients with Parkinson's disease receiving dopamine replacement therapy: evidence and implications for the addictions field. Addiction.

[CR2] Baig F, Kelly MJ, Lawton MA, Ruffmann C, Rolinski M, Klein JC, Barber T, Lo C, Ben-Shlomo Y, Okai D, Hu MT (2019). Impulse control disorders in Parkinson disease and RBD: a longitudinal study of severity. Neurology.

[CR3] Becker S, Bäumer A, Maetzler W, Nussbaum S, Timmers M, Van Nueten L, Salvadore G, Zaunbrecher D, Roeben B, Brockmann K, Streffer J, Berg D, Liepelt-Scarfone I (2020). Assessment of cognitive-driven activity of daily living impairment in non-demented Parkinson's patients. J Neuropsychol.

[CR4] Cao L, Xu T, Zhao G, Ly D, Lu J, Zhao G (2022). Risk factors of impulsive-compulsive behaviors in PD pateints: a meta-analysis. J Neurology.

[CR5] Corvol J-C, Artaud F, Cormier-Dequaire F, Rascol O, Durif F, Derkinderen P, Marques A-R, Bourdain F, Brandel J-P, Pico F, Lacomblez L, Bonnet C, Brefel-Courbon C, Ory-Magne F, Grabli D, Klebe S, Mangone G, You H, Mesnage V, Lee P-C, Brice A, Vidailhet M, Elbaz A (2018). Longitudinal analysis of impulse control disorders in Parkinson disease. Neurology.

[CR6] Dagher A, Robbins TW (2009). Personality, addiction, dopamine: insights from Parkinson’s disease. Neuron.

[CR7] De Wit LE, Wilting I, Souverein PC, van der Pol P, Egberts TCG (2022). Impulse control disorders associated with dopaminergic drugs: a disproportionality analysis using vigibase. Eur Neuropsychopharmacol.

[CR8] Delaney M, Leroi I, Simpson J, Overton PG (2012). Impulse control disorders in Parkinson’s disease: a psychosocial perspective. J Clin Psychol Med Settings.

[CR9] Djamshidian A, Jha A, O'Sullivan SS, Silveira-Moriyama L, Jacobson C, Brown P, Lees A, Averbeck BB (2010). Risk and learning in impulsive and nonimpulsive patients with Parkinson’s disease. Mov Disord.

[CR10] Djamshidian A, O'Sullivan SS, Doherty K, Lees AJ, Averbeck BB (2011). Altruistic punishment in patients with Parkinson’s disease with and without impulsive behaviour. Neuropsychologia.

[CR11] Djamshidian A, O'Sullivan SS, Wittmann BC, Lees AJ, Averbeck BB (2011). Novelty seeking behaviour in Parkinson’s disease. Neuropsychologia.

[CR12] Emre M, Aarsland D, Brown R, Burn DJ, Duyckaerts C, Mizuno Y, Broe GA, Cummings J, Dickson DW, Gauthier S, Goldman J, Goetz C, Korczyn A, Lees A, Levy R, Litvan I, McKeith I, Olanow W, Poewe W, Quinn N, Sampaio C, Tolosa E, Dubois B (2007). Clinical diagnostic criteria for dementia associated with Parkinson’s disease. Mov Disord.

[CR13] Ferrara JM, Stacy M (2008). Impulse-control disorders in Parkinson’s disease. CNS Spectr.

[CR14] Goetz CG, Tilley BC, Shaftman SR, Stebbins GT, Fahn S, Martinez-Martin P, Poewe W, Sampaio C, Stern MB, Dodel R, Dubois B, Holloway R, Jankovic J, Kulisevsky J, Lang AE, Lees A, Leurgans S, LeWitt PA, Nyenhuis D, Olanow CW, Rascol O, Schrag A, Teresi JA, van Hilten JJ, LaPelle N (2008). Movement disorder society-sponsored revision of the unified Parkinson’s disease rating scale (MDS-UPDRS): scale presentation and clinimetric testing results. Mov Disord.

[CR15] Grall-Bronnec M, Victorri-Vigneau C, Donnio Y, Leboucher J, Rousselet M, Thiabaud E, Zreika N, Derkinderen P, Challet-Bouju G (2018). Dopamine agonists and impulse control disorders: a complex association. Drug Saf.

[CR16] Grüsser SM, Thalemann CN (2006). Verhaltenssucht: Diagnostik, Therapie, Forschung [Behavioural addiction: Diagnostics, therapy, research].

[CR17] Harris PA, Taylor R, Thielke R, Payne J, Gonzalez N, Conde JG (2009). Research electronic data capture (REDCap): a metadata-driven methodology and workflow process for providing translational research informatics support. J Biomed Inform.

[CR18] Hautzinger M, Keller F, Kühner C (2009) Beck Depressions-Inventar Revision (BDI II). Pearson Frankfurt

[CR19] Hughes AJ, Daniel SE, Kilford L, Lees AJ (1992). Accuracy of clinical diagnosis of idiopathic Parkinson’s disease: a clinico-pathological study of 100 cases. J Neurol Neurosurg Psychiatry.

[CR20] Jiménez-Murcia S, Bove FS, Israel M, Steiger H, Fernandez-Aranda F, Álvarez-Moya E, Granero R, Penelo E, Vergé B, Aymami M-N, Santamaria JJ, Gómez-Pna M, Moragas L, Daccidou LG, Menchón JM (2012). Cognitive behavioral therapy for pathological gambling in Parkinson's disease: a pilot controlled study. Eur Addict Res.

[CR21] Leplow B, Sepke M, Schönfeld R, Pohl J, Oelsner H, Latzko L, Ebersbach G (2017). Impaired learning of punishments in Parkinson’s disease with and without impulse control disorder. J Neural Transm.

[CR22] Limotai N, Oyama G, Go C, Bernal O, Ong T, Moum SJ, Bhidayasiri R, Foote KD, Bowers D, Ward H, Okun MS (2012). Addiction-like manifestations and Parkinson’s disease: a large single center 9-year experience. Int J Neurosci.

[CR23] Marin-Lahoz J, Pagonabarraga J, Martinez-Horta S, Fernandez de Bobadilla R, Pascual-Sedano B, Pérez-Pérez J, Gironell A, Kulisevsky J (2018). Parkinson’s disease: impulsivity does not cause impulse control disorders but boosts their severity. Front Psychiatry.

[CR24] Marín-Lahoz J, Martinez-Horta S, Pagonabarraga J, Horta-Barba A, Aracil-Bolaños I, Bejr-kasem H, Sampedro F, Campolongo A, Kulisevsky J (2022). Predicting Impulse control disorders in Parkinson disease through incentive biomarkers. Ann Neurol.

[CR25] Marques A, Durif F, Fernagut P-O (2018). Impulse control disorders in Parkinson’s disease. J Neural Transm.

[CR26] Nagelkerke NJD (1991). A note on a general definition of the coefficient of determination. Biometrika.

[CR27] Nasreddine ZS, Phillips NA, Bédirian V, Charbonneau S, Whitehead V, Collin I, Cummings JL, Chertkow H (2005). The Montreal cognitive assessment, MoCA: a brief screening tool for mild cognitive impairment. J Am Geriatr Soc.

[CR28] Navalpotro-Gomez I, Kim J, Paz-Alonso PM, Delgado-Alvarado M, Quiroga-Varela A, Jimenez-Urbieta H, Carreiras M, Strafella AP, Cruz Rodriguez-Oroz M (2020). Disrupted salience network dynamics in Parkinson's disease patients with impulse control disorders. Parkinsonism Relat Disord.

[CR29] Okai D, Askey-Jones S, Samuel M, O'Sullivan SS, Chaudhuri KR, Martin A, Mack J, Brown RG, David AS (2013). Trial of CBT for impulse control behaviors affecting Parkinson patients and their caregivers. Neurology.

[CR30] O'Sullivan SS, Wu K, Politis M, Lawrence AD, Evans AH, Bose SK, Djamshidian A, Lees AJ, Piccini P (2011). Cue-induced striatal dopamine release in Parkinson’s disease-associated impulsive-compulsive behaviours. Brain.

[CR31] Peto V, Jenkinson C, Fitzpatrick R (1998). PDQ-39: A review of the development, validation and application of a Parkinson’s disease quality of life questionnaire and its associated measures. J Neurol.

[CR32] Phu AL, Xu Z, Brakoulias V, Mahant N, Fung VSC, De Moore G, Martin A, Starcevic V, Krause M (2014). Effect of impulse control disorders on disability and quality of life in Parkinson’s disease patients. J Clin Neurosci.

[CR33] Probst CC, van Eimeren T (2013). The functional anatomy of impulse control disorders. Curr Neurol Neurosci Rep.

[CR34] R Core Team (2021) R (Version 4.1.2) [Computer software]. https://www.R-project.org.

[CR35] Ramdave S, Dawson A, Carter A, Dissanayaka NNW (2020). Unmasking neurobiological commonalities between addictive disorders and impulse control disorders in Parkinson’s disease. Brain Imaging Behav.

[CR36] Schade S, Mollenhauer B, Trenkwalder C (2020). Levodopa equivalent dose conversion factors: An updated proposal including opicapone and safinamide. Mov Disord Clin Pract.

[CR37] Storch A, Odin P, Trender-Gerhard I, Fuchs G, Reifschneider G, Ray Chaudhuri K, Jost WH, Ebersbach G (2010). Non-motor symptoms questionnaire and scale for Parkinson’s disease: cross-cultural adaptation into the german language. Nervenarzt.

[CR38] Tomer R, Aharon-Peretz J (2004). Novelty seeking and harm avoidance in Parkinson’s disease: Effects of asymmetric dopamine deficiency. J Neurol Neurosurg Psychiatry.

[CR39] Tomlinson CL, Stowe R, Patel S, Rick C, Gray R, Clarke CE (2010). Systematic review of levodopa dose equivalency reporting in Parkinson's disease. Mov Disord.

[CR40] Voon V, Fox SH (2007). Medication-related impulse control and repetitive behaviors in Parkinson disease. Arch Neurol.

[CR41] Voon V, Pessiglione M, Brezing C, Gallea C, Fernandez HH, Dolan RJ, Hallett M (2010). Mechanisms underlying dopamine-mediated reward bias in compulsive behaviors. Neuron.

[CR42] Voon V, Reynolds B, Brezing C, Gallea C, Skaljic M, Ekanayake V, Fernandez H, Potenza MN, Dolan RJ, Hallett M (2010). Impulsive choice and response in dopamine agonist-related impulse control behaviors. Psychopharmacology.

[CR43] Weintraub D, Claassen DO (2017). Impulse control and related disorders in Parkinson’s disease. Int Rev Neurobiol.

[CR44] Weintraub D, Nirenberg MJ (2013). Impulse control and related disorders in Parkinson’s disease. Neurodegener Dis.

[CR45] Weintraub D, Siderowf AD, Potenza MN, Goveas J, Morales KH, Duda JE, Moberg PJ, Stern MB (2006). Association of dopamine agonist use with impulse control disorders in Parkinson disease. Arch Neurol.

[CR46] Weintraub D, Hoops S, Shea JA, Lyons KE, Pahwa R, Driver-Dunckley ED, Adler CH, Potenza MN, Miyasaki J, Siderowf AD, Duda JE, Hurtig HI, Colcher A, Horn SS, Stern MB, Voon V (2009). Validation of the questionnaire for impulsive-compulsive disorders in Parkinson’s disease. Mov Disord.

[CR47] Weintraub D, Koester J, Potenza MN, Siderowf AD, Stacy M, Voon V, Whetteckey J, Wunderlich GR, Lang AE (2010). Impulse control disorders in Parkinson disease: a cross-sectional study of 3090 patients. Arch Neurol.

[CR48] Weintraub D, Papay K, Siderowf A (2013). Screening for impulse control symptoms in patients with de novo Parkinson disease: a case-control study. Neurology.

[CR49] Weintraub D, David AS, Evans AH, Grant JE, Stacy M (2015). Clinical spectrum of impulse control disorders in Parkinson's disease. Mov Disord.

[CR50] Weintraub D, Posavi M, Fontanillas P, Tropea TF, Mamikonyan E, Suh E, Trojanowski JQ, Cannon P, Van Deerlin VM, 23andMe Research Team, Chen-Plotkin AS (2022). Genetic prediction of impulse control disorders in Parkinson‘s disease. Ann Clin Transl Neurol.

